# Multiple Locus Linkage Analysis of Genomewide Expression in Yeast

**DOI:** 10.1371/journal.pbio.0030267

**Published:** 2005-07-26

**Authors:** John D Storey, Joshua M Akey, Leonid Kruglyak

**Affiliations:** **1**Department of Biostatistics, University of Washington, Seattle, Washington, United States of America,; **2**Department of Genome Sciences, University of Washington, Seattle, Washington, United States of America,; **3**Department of Ecology and Evolutionary Biology, Princeton University, Princeton, New Jersey, United States of America,; **4**Lewis-Sigler Institute for Integrative Genomics, Princeton University, Princeton, New Jersey, United States of America; University of OxfordUnited Kingdom

## Abstract

With the ability to measure thousands of related phenotypes from a single biological sample, it is now feasible to genetically dissect systems-level biological phenomena. The genetics of transcriptional regulation and protein abundance are likely to be complex, meaning that genetic variation at multiple loci will influence these phenotypes. Several recent studies have investigated the role of genetic variation in transcription by applying traditional linkage analysis methods to genomewide expression data, where each gene expression level was treated as a quantitative trait and analyzed separately from one another. Here, we develop a new, computationally efficient method for simultaneously mapping multiple gene expression quantitative trait loci that directly uses all of the available data. Information shared across gene expression traits is captured in a way that makes minimal assumptions about the statistical properties of the data. The method produces easy-to-interpret measures of statistical significance for both individual loci and the overall joint significance of multiple loci selected for a given expression trait. We apply the new method to a cross between two strains of the budding yeast *Saccharomyces cerevisiae,* and estimate that at least 37% of all gene expression traits show two simultaneous linkages, where we have allowed for epistatic interactions. Pairs of jointly linking quantitative trait loci are identified with high confidence for 170 gene expression traits, where it is expected that *both* loci are true positives for at least 153 traits. In addition, we are able to show that epistatic interactions contribute to gene expression variation for at least 14% of all traits. We compare the proposed approach to an exhaustive two-dimensional scan over all pairs of loci. Surprisingly, we demonstrate that an exhaustive two-dimensional scan is less powerful than the sequential search used here. In addition, we show that a two-dimensional scan does not truly allow one to test for simultaneous linkage, and the statistical significance measured from this existing method cannot be interpreted among many traits.

## Abstract 2

Complex traits are frequently under control of multiple loci. This new method simultaneously maps multiple gene expression quantitative trait loci and assesses their significance within *Saccharomyces cerevisiae.*

## Introduction

Genetic linkage analysis has traditionally been applied to one or very few traits at a time. It is now possible to simultaneously measure thousands of related “traits” from high-throughput technologies such as DNA [1,2] and protein microarrays [3]. It is therefore necessary to extend linkage analysis techniques so that thousands of traits can be simultaneously analyzed, particularly when the traits are complex and it is desirable to identify multiple loci contributing to phenotypic variation. Performing linkage analysis on many traits compounds difficulties that are already present in a conventional analysis [4], although it also provides the opportunity to borrow information across the traits in such a way that more informative conclusions can be drawn. Here, we focus on linkage analysis of genomewide expression data obtained from DNA microarrays. Recent studies in a variety of organisms [5–13] have unambiguously shown that heritable variation in gene expression levels is pervasive. Therefore, there is considerable interest in delineating the genetic architecture of transcriptional variation at the genomewide level.

Existing linkage analysis techniques have already been applied to genomewide expression in yeast, mice, maize, and humans [5–7]. In a cross between two strains of the budding yeast *Saccharomyces cerevisiae,* linkage scans were performed separately on the expression levels of approximately 6,000 genes. It was shown that hundreds of these “gene expression traits” show linkage to at least one locus, and many traits appear to be influenced by multiple quantitative trait loci (QTL) [5,7,14]. However, multiple locus linkage analysis has not been applied to this dataset. A well accepted approach for mapping two loci, and for identifying epistatic interactions, is to perform an exhaustive two-dimensional (2D) linkage analysis in which all pair-wise positions in the genome are tested for linkage. However, 2D scans are extremely computationally demanding when applied to thousands of phenotypes and may suffer from low statistical power due to the large number of hypothesis tests performed.

Although several other approaches exist for mapping multiple loci that are linked to a quantitative trait, none of these methods allows the individual and *joint* significance of the loci to be unequivocally assessed. Here, the joint significance is the case where *all* of the multiple loci are truly linked, not just a subset of them. Thresholding based on the joint significance becomes particularly important when examining many quantitative traits at once. One common approach for identifying multiple QTL is to use a model selection algorithm [15–18], where the goal is to identify the subset of loci comprising the best model according to some optimality criterion. Using this approach, it is difficult to search over the enormous number of potential models and to provide a criterion for the best model that is biologically meaningful. Furthermore, for models that include multiple QTL, it is difficult to determine which of these are true QTL and which have been included in the model by chance. Another approach is to *a priori* form a model that includes a certain set of pre-chosen loci spanning the genome, and then test whether each remaining locus significantly improves the model in this larger multiple locus model [19–23]. The motivation for this mapping approach and its derivatives such as multiple interval mapping is that the pre-chosen loci explain residual variation of other unknown QTL. It is difficult to formulate a pre-chosen model for each expression trait, and again it is difficult to interpret the joint significance among multiple loci.

To address these issues, we have developed a new method for mapping multiple loci and identifying epistatic interactions when analyzing thousands of phenotypes, such as gene expression levels. Information shared across expression traits is employed in a way that allows us to make minimal assumptions about the statistical properties of the data. Our method permits easy-to-interpret statistical significance analysis of individual loci, as well as the overall joint significance of multiple loci identified for any given expression trait. Strengths of both the model selection and composite interval mapping methods have been incorporated, which turns out to be more straightforward when analyzing many traits simultaneously. Rather than trying to estimate the true model underlying the expression trait by seeking the “best model,” or by assuming a certain model of genetic background and testing for the inclusion of additional loci, we propose to measure *the probability that a locus is in the true model* given the data, without ever specifically estimating the entire true model. This overcomes some of the difficulties incurred by applying the two existing approaches to this problem. However, if one were considering only a single trait, it would be very difficult to calculate this probability without making strong assumptions. Here, we use a nonparametric approach that allows us to calculate conservative estimates of these posterior probabilities of linkage. The nonparametric approach is possible because many related traits are considered simultaneously.

We applied the method to the *S. cerevisiae* experiment and show that at least 37% of all gene expression traits show joint linkage to two loci. Pairs of jointly linking QTL could be identified with high confidence for 170 gene expression traits. Bioinformatics analysis of these 170 significant expression traits and their corresponding QTL begins to provide intriguing insights into the genetic architecture of transcriptional variation. In addition, we are able to show that epistatic interactions contribute to gene expression variation in at least 14% of all traits. Our proposed approach overcomes the inherent computational and statistical difficulties that arise when performing an exhaustive search on thousands of traits at once. Moreover, the availability of thousands of traits for a single set of meioses allows us to show that, for this experiment, a full 2D scan is not as powerful as the sequential search method we employ, even though the locus pairs selected by the two methods overlap substantially. We also show that 2D scans do not allow one to test for joint linkage of a pair of loci, only whether at least one of the pair is linked. This is shown to be particularly problematic when analyzing thousands of phenotypes.

## Results

The data used in this study were derived from a cross between two haploid strains of the budding yeast *S. cerevisiae*: a standard laboratory strain and a wild isolate from a California vineyard. Gene expression measurements were obtained for 6,216 open reading frames in 112 haploid segregants, and dichotomous genotypes were identified at 3,312 markers covering 99% of the genome [14]. Using these experimental data, we developed and applied a new computationally efficient method for simultaneously mapping multiple gene expression QTL and identifying epistatic interactions. The models and methods used here are appropriate for haploid organisms, although the ideas may be extended to diploid and higher ploidy organisms in the usual way [24].

### 2D Linkage Scan

Initially, we applied an exhaustive 2D linkage scan in order to identify expression traits that are significantly linked to pairs of loci or that are significant for epistasis. In performing these significance tests, we considered a linear model that fully parameterizes the quantitative trait in terms of all four possible genotypes. This model can be written as



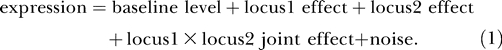



Traditionally, genetic linkage is said to exist between the trait and a pair of loci if any of the locus effects are significantly different than zero, but not necessarily all of them [24]. Epistasis exists only if the locus1 × locus2 interaction is significantly different from zero. This linear model approach to identifying QTLs is well justified [24] and has been shown to be especially useful when the markers are densely sampled [18].

The test for pair-wise linkage was performed as follows. For each expression trait, a linear model was fit by least squares to each pair of loci. The locus pair with the largest F-statistic comparing the full model to the baseline model was selected for that trait. For the test of epistasis, a similar procedure was performed, except an F-statistic was computed that compared the full model to the purely additive model, which directly assesses the contribution of the interaction term. The significance of each locus pair selected for linkage was computed using a standard permutation technique against the null hypothesis of no linkage to either locus [25]. For the test of epistasis, we used a similar permutation technique to assess the significance of the locus1 × locus2 interaction [26]. The end-product of these tests is a *p-*value and a pair of loci for each expression trait. Using the false discovery rate (FDR) quantity to correct for multiple comparisons [27,28], there were 3,540 traits significantly linked to a locus pair at the 5% FDR threshold. Note that in this case, a “false discovery” must be defined as a trait where both selected loci are false positives, and thus many of the “true discovery” traits could have one false-positive locus. In the test for epistasis, no significant results were obtained; the number of significant tests at each cut-off mirrored the number found under the null permutations.

The exhaustive 2D search proved to be unsatisfactory for a number of reasons. Most obviously, the number of multiple-locus models that have to be considered is computationally and statistically challenging for pairs of loci, and prohibitive for three or more loci. With 3,312 markers and 6,216 expression traits, one has to consider more than 18 million single-locus models to simply test for linkage between every expression trait and locus. More than 27 billion tests have to be performed to consider all two-locus models for every expression trait, and more than 27 trillion tests to consider all three-locus models for every expression trait. In addition, it is likely that by searching over so many models, the statistical power to detect linkage is severely attenuated because of the multiple comparison problem. Secondly, when employing an exhaustive 2D scan, there is no statistically rigorous method to test for joint linkage, which exists only if both loci have nonzero terms in the full model. In other words, the significance of an individual locus selected for an expression trait is confounded with the overall significance of the pair of loci. Since *p-*values are calculated against the null hypothesis of no linkage, a highly significant result may be due to only one locus being truly linked while the other locus is included by chance. This confounding is especially problematic when considering thousands of traits simultaneously. Since pairs of loci that show large marginal effects are preferentially selected when testing thousands of traits for linkage, one cannot examine marginal effects of individual loci among the most significant linkages in an unbiased fashion. Therefore, by chance it may appear that both loci explain a large proportion of variance of the trait. Ideally, a measure of significance for each locus would be available, and then a joint measure of significance for all loci would be calculated. In our case, we did not want to call a trait significantly linked if *either* of the loci were a false positive. Finally, a decision must be made as to which traits to call significantly linked. If the goal is to avoid any false-positive loci when calling a trait significant, then there is no simple *p-*value that can be formed for this purpose. This follows because the null hypothesis consists of multiple scenarios, and there is no readily available null distribution to describe this. Therefore, a more sophisticated method must be used to assess the significance of thousands of multi-locus models.

### Stepwise Search More Powerful than 2D Search

One potential way to improve the exhaustive 2D scan is to use another method for selecting pairs of loci. In particular, one can select loci in a sequential manner, cutting down the number of models considered to 2 × 3,312, instead of more than 5 million. One readily available method for selecting loci in a sequential manner is forward stepwise regression. Here, one selects a primary locus that shows the most significant one-dimensional (1D) linkage, i.e., the one that has the largest LOD score. This is equivalent to identifying the locus that yields the smallest residual sum of squares when regressing the expression trait on the inheritance pattern at that locus [24]. Next, a secondary locus is chosen that yields the largest LOD score conditional on the primary locus being linked. Again, this is equivalent to choosing a locus that minimizes the residual sum of squares when regressing the expression trait on both the secondary locus and the primary locus contained in the same model. A tertiary locus may be selected by including the previous two loci in the regression, and so on.

**Figure 1:**
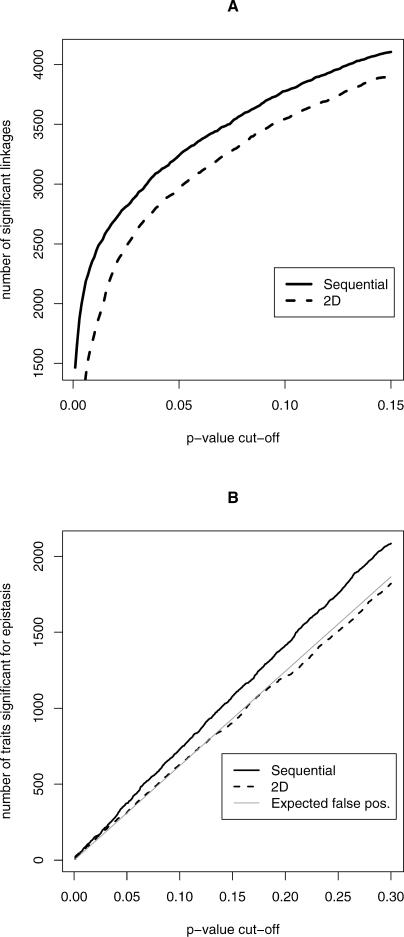
A Power Comparison of the 2D Locus Pair Search and the Sequential Search The number of significant traits over a range of *p-*value thresholds are shown. Since any given *p-*value threshold results in the same number of expected false positives, these plots give empirical evidence that the sequential search is more powerful than the 2D search. (A) Plot of the number of traits significant for linkage versus the *p-*value threshold. (B) Plot of the number of traits significant for epistasis versus the *p-*value threshold. The gray line, which shows the number of expected false positives for each *p-*value cut-off, is similar to the number called significant under the exhaustive 2D search.

One can use this forward stepwise regression technique simply as a way to select pairs of loci for each trait, and then the significance analysis can be repeated as before. It has been hypothesized that failing to consider all possible two-locus models through an exhaustive 2D search (e.g., selecting loci in a sequential fashion) may lead to a loss in power or to missing important interactions between loci [29]. The yeast expression dataset presents a rare opportunity to test this idea; in a typical study, only one quantitative trait is measured and only one scan is performed, which makes it difficult to compare different multi-locus selection methods. Simulation-based comparisons make a number of assumptions that may not always be true. Here, we are able to make a direct comparison based on thousands of related scans, all conditional on the same set of meioses. Thus, we compared the exhaustive 2D search to a simple sequential search method by repeating every step exactly as above, except that the sequential search method was used to select pairs of loci (see Materials and Methods). At FDR cut-offs of 1% and 5%, there are 2,780 and 4,271 significant pair-wise linkages, respectively. At these same cut-offs, the 2D search yielded substantially fewer: 1,715 and 3,540 significant linkages. Figure 1A shows the number of significant pair-wise linkages over a range of *p-*value thresholds, where it can be seen that the sequential scan consistently finds a greater number. Since any given *p-*value threshold results in the same number of expected false positives for each type of search, this is empirical evidence that the sequential search is more powerful. A 2D search could still produce more biologically meaningful results. In order to assess this, we measured the overlap in selected locus pairs among the traits corresponding to the 3,000 most significant linkages identified by the 2D method. The locus pairs selected by the two methods for each given trait were considered to be equivalent if the same two chromosomes were identified and the respective chromosomal locations of the loci were within 50 kilobases (kb) of each other. Under this definition, 90% of the locus pairs were found to be equivalent between the two methods.

The sequential search was also more powerful for identifying epistasis relative to the exhaustive 2D scan. Figure 1B shows the number of traits called significant for epistasis over a range of *p-*value thresholds, where it can be seen that the sequential search is again more powerful. Neither search method yielded a trait with high significance for epistasis. However, from the sequential method we are able to estimate that at least 14% of the traits are operating under epistasis, whereas due to a lack of power the 2D search estimate is 0%. This estimate is obtained by the following reasoning, which has been rigorously developed elsewhere [27,28]. If the locus pair identified for each transcript were a false positive, the distribution of *p-*values across all transcripts would be flat and uniformly distributed between zero and one; thus, the shape of the observed distribution of *p-*values can be used to estimate the total proportion of false positives. The more powerful a set of statistical tests are, the more this flatness can be distinguished from the signal. We did not see much overlap in locus pairs selected among the two search methods, but this is not surprising given that the 2D search apparently produces only noise.

Therefore, for this particular experiment, the sequential search is more powerful than the exhaustive 2D search in identifying pair-wise linkage and detecting epistasis. The sequential search also appears to extract a biological signal that is similar to that from the 2D search. However, it is not possible to conclude whether these properties would hold in other experiments or for different sample sizes. Also, the comparison was made based on significance assessed against the null hypothesis of no linkage, which is not a solution to the problem of detecting joint linkage. The sequential approach as implemented above still suffers from the problem that significance can be driven by a single locus while the other locus is a false positive. However, sequentially selecting loci allows their individual significance to be assessed, which we show is crucial in detecting true joint linkage. We discuss how to assess individual and joint significance for the sequential approach below; we note that the same methods would not work without a number of potentially unjustifiable assumptions for the exhaustive 2D search.

### Proposed Approach

We developed a method to overcome the following problems associated with existing approaches: a prohibitively large number of multi-locus models are considered, a clear measure of significance among individual loci is not available, and the desired alternative hypothesis that *all* selected loci are linked for each trait is not tested. The method can be summarized in four steps.

**Step 1.** For each expression trait,*L* loci are identified through a sequential locus selection procedure, as above.

**Step 2.** At each stage of the sequential search, a Bayesian technique is employed to calculate the probability that the locus is linked to the expression trait, conditional on the assumption that the previously chosen loci are also linked.

**Step 3.** The locus-specific probabilities are combined to form the probability that all loci are simultaneously linked to the expression trait.

The overall probabilities of linkage from Step 3 provide a ranking of the traits from most significant to least significant. It is then necessary to select a set of traits, each of which has a high probability of being linked to all loci simultaneously. In order to guide this choice, we propose a method to assess the statistical significance of a given set of traits.

**Step 4.** A significant expression trait is called a false discovery if any of the loci selected for that trait is a false positive. That is, a true discovery is an expression trait where *all* selected loci are truly linked. A new approach for estimating the FDR among a set of significant traits is employed that directly utilizes the probabilities calculated in Step 3.

The starting point for the method is to define a multi-locus model that may include varying numbers of loci, where it is clear how one modifies the model to include an additional locus. Here, we continue to use the fully parameterized model. For zero, one, and two loci, the model may be written, respectively, as



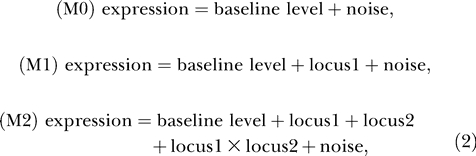



where, for example, “locus1” is the main effect for the primary locus, and “locus1 **×** locus2” is the epistatic interaction between the primary and secondary loci (Materials and Methods). A sequential search can then be performed to identify the top linked locus for each expression trait, which involves finding the locus that offers the greatest improvement in goodness of fit when comparing model M1 to model M0. This primary locus is then included in models M1 and M2, and a secondary locus is identified that provides the greatest improvement in goodness of fit when comparing model M2 to model M1. Continuing this process, an ordered set of *L* loci for each expression trait can be identified. Here we consider only *L* = 2 loci, but the method can be applied to larger numbers of loci.

The Bayesian posterior probability that the primary locus for each trait shows linkage can be written as **Pr**(locus 1 linked |Data). Since the secondary locus is identified conditional on the presence of the primary locus, the probability that it is linked is calculated conditionally on the primary locus being linked: **Pr**(locus 2 linked | locus 1 linked, Data). Note that probabilities may be formed analogously for *L* loci, with the final probability being**Pr**(locus L linked |loci 1,...,L-1 linked, Data). These conditional probabilities are conceptually consistent with the procedure used to select loci. For example, the secondary locus is not called significant unless the primary locus is also called significant, since it was used in identifying the secondary locus.

The above probabilities give a measure of significance to each locus. However, one would also like to know the *joint* significance of the loci. The probability that *all* loci are linked to the expression trait is simply the product of the locus-specific probabilities. For example,



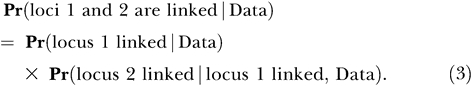



These joint-linkage probabilities can be used to select traits that are significant for having all loci jointly linked by calling all traits significant that have a joint-linkage probability exceeding some threshold. For example, all traits with **Pr**(loci 1 and 2 are linked | Data) ≥ 0.90 may be called significant. This threshold is equivalent to ranking the traits for significance by the size of the joint-linkage probability. (Variations on this ranking procedure are possible, depending on the goals of the study; e.g., one may want to consider only traits that have all locus-specific linkages probabilities at 0.95 or greater—see Materials and Methods.) In order to decide on a reasonable threshold, an error rate associated with the thresholding rule is assessed. For example, how reliable is the list of traits that have a joint-linkage probability of 0.90 or greater? The FDR concept is attractive in this case since thousands of traits are simultaneously being assessed, and we would like to select several without incurring too many mistakes. The FDR is typically defined and estimated in terms of multiple hypothesis tests [27,28]. However, the “null hypothesis” here is complicated because it includes any scenario where one or both of the loci are not truly linked. However, when assuming a Bayesian model, the FDR has been shown to be equal to a Bayesian posterior probability [30], and the FDR concept and estimation methodology can be extended to accommodate our situation.

A trait is defined to be a “false discovery” for joint linkage if any of its selected loci is a false positive. In standard multiple hypothesis testing situations, the false discovery has been estimated as the ratio of the estimated number of false positive divided by the observed number of tests called significant. For a given threshold we place on the traits, it is straightforward to count how many are called significant, but it is not as easy to estimate the expected number of false positives because the null distribution of the joint-linkage probabilities is not available. However, when identifying pairs of loci for each trait, the probability a trait is a false discovery is 1 −**Pr**(loci 1 and 2 are linked| Data). Therefore, the overall expected number of false discoveries is the sum of the 1 −**Pr**(loci 1 and 2 are linked| Data) over all traits called significant for two-locus linkage. An estimate of the proportion of false discoveries among significant linkages is then



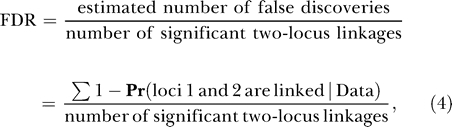



where again the summation is taken over all traits called significant for a two-locus linkage. This estimate can be justified in the context of Bayesian representations of the FDR, but it also has connections to *p-*value based estimates ([30]; Materials and Methods). For example, in our study there are 72 traits that have two-locus joint-linkage probabilities of 0.90 or greater. Summing all 72 corresponding quantities 1−**Pr** (loci 1 and 2 are linked | Data), there are 4.8 expected false discovery two-locus linkages among these. Therefore, the FDR estimate of this particular threshold is 4.8/72 = 6.7%.

In practice, the locus-specific and joint-linkage probabilities must be estimated. Due to the massive amount of available data, we form nonparametric estimates of the probabilities rather than making assumptions about their distributions. At each stage of the locus selection, the strength of linkage is quantified by a standard F-statistic used to compare two models (M1 versus M0 or M2 versus M1). The statistics associated with the primary and secondary loci for each trait are the maximal F-statistics among all loci. Since these maximal statistics do not have a known null distribution, the null distributions are simulated. The quantitative trait values are permuted and the maximal statistics are recomputed [25] to give permutation null statistics. Note that when the null statistics are simulated for the secondary loci, the fact that the primary loci are assumed to be truly linked is taken into account [26]. That is, the null statistics corresponding to the secondary loci are calculated conditionally on the genotypes of the primary loci. We performed five permutations to yield sets of 6,216 **×** 5 simulated null statistics corresponding to the primary and secondary locus selections. The observed statistics and null statistics corresponding to the primary loci are used to estimate the linkage probabilities **Pr**(locus 1 linked | Data) for each trait. Similarly, the observed and null statistics corresponding to the secondary loci are used to estimate **Pr**(locus 2 linked | locus 1 linked, Data) for each trait.

**Figure 2:**
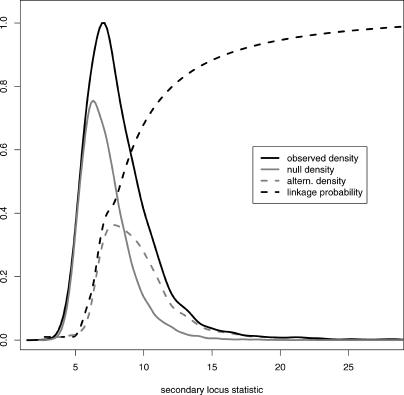
An Example of the Locus-Specific Linkage Probability Estimation Applied to the Secondary Loci The estimated density of the observed statistics is plotted (solid black). This density is modeled as a weighted mixture of probability densities corresponding to the “null” unlinked secondary loci (solid grey) and the “alternative” linked secondary loci (dashed grey). The estimated posterior probability of linkage is also shown (dashed black).

A key aspect of our proposed approach is that loci are selected one at a time for each given expression trait. In the traditional approach, pairs of loci are selected together so that among these locus pairs, zero, one, or two loci may be truly linked. Therefore, it is not possible to model all three cases without making a number of assumptions. However, since we select only one locus at a time, there are only two possible outcomes at each selection step: the locus is either linked or not. The statistics calculated at each locus selection stage are a mixture of the two distributions corresponding to these linked and unlinked loci. The permutation null statistics represent one component of this mixture and can be used in conjunction with the observed statistics to conservatively estimate the locus-specific linkage probabilities. Figure 2 shows a plot of the strategy used to form these estimates. The solid black line is an empirical probability density (i.e., smoothed histogram) of the 6,216 observed statistics calculated from the secondary loci. This density is a mixture of a null density corresponding to statistics of unlinked loci, and an alternative density corresponding to statistics of linked loci. The solid grey line is an empirical probability density of the permutation null statistics, which has been drawn to reflect its relative contribution to the black mixture density of observed statistics. The observed statistics and permutation null statistics can be used to conservatively estimate the proportion of true linkages among all secondary loci ([27]; Materials and Methods), which is the mixing proportion of the null density and the prior probability of linkage. In order to calculate the locus-specific linkage probability, the ratio of the null density to the mixture density must also be estimated. This ratio is estimated by adaptively considering the relative frequency of null statistics to observed statistics in small intervals around each possible value of the observed statistics (Materials and Methods). Once the locus-specific linkage probabilities are estimated, these quantities are plugged into the above proposed procedure to obtain a set of significant two-locus linkages.

### Two-Locus Joint Linkage Applied to Gene expression Traits in Yeast

We applied the proposed method for two-locus linkage analysis to the *S. cerevisiae* experiment. Based on the joint-linkage probabilities, we estimate that 2,300 traits (approximately 37%) are jointly linked to two loci, although we cannot identify all of these with high confidence. Of these 2,300 traits, 170 can be identified at a FDR of 10%. Among these 170 traits, the primary locus FDR is less than 0.2%. Therefore, we expect that at most 17 of these joint linkages include a single false-positive locus, and about zero include two false-positive loci.

Recall that when a more liberal definition of two-locus linkage was used, where only one locus was required to be linked, about 4,000 linkages were called significant at a FDR of 5%. However, in that situation it was not clear whether both loci or just a single locus were truly linked. Because we identify only 170 significant joint linkages at a FDR of 10%, it appears that many of the 4,000 significant linkages from the other approach were due to only a single locus being truly linked. When comparing our method to a traditional 1D linkage scan where the top two linkage peaks are taken as significant, we find 3.3 to 8.7 times more linkages at FDR cut-offs ranging from 1% to 10% (Materials and Methods). These observations indicate that our proposed approach provides a new and statistically rigorous framework for distinguishing between genetic models.

To better understand the molecular mechanisms underlying the observed linkages for these traits, we searched for *cis*-acting effects. Here a *cis* linkage is said to occur if one of the two linkage peaks coincides with the position of the encoding gene corresponding to the expression trait. In total, 58 traits demonstrate a *cis* linkage (Table S1), which has two important implications. First, the observation of a *cis* linkage immediately suggests a candidate QTL that can be experimentally tested. Second, these results demonstrate that variation in the expression level for a given trait cannot simply be dichotomized into either *cis* or *trans* effects, as both can simultaneously contribute to variation in gene expression levels.

**Figure 3:**
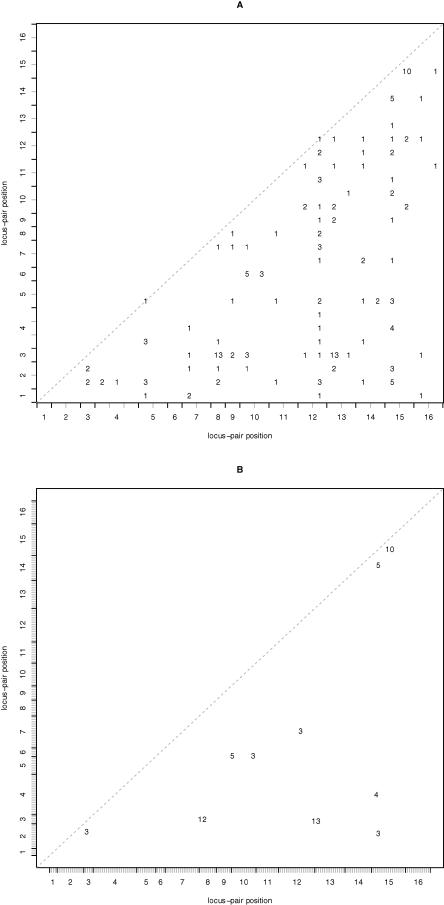
A Plot of the Locus Pair Positions Corresponding to the 170 Traits Significant for Joint Linkage (A) A plot of the significant locus pair positions when each chromosome has been partitioned into equally sized bins less than or equal to 550 kb. The number of significant traits showing linkage to locus pairs in each pair-wise bin is denoted. The number on each axis indicates the chromosome number; a dash denotes a bin division. (B) A plot constructed analogously to (A), except bins less than or equal to 50 kb are used, and only bins with three or more traits significant for joint linkage are numbered.

Several previous linkage analyses of gene expression levels in yeast and other organisms have shown that linkages are nonrandomly distributed throughout the genome and tend to cluster into specific locations [5-7,31]. In order to get a broad view of the distribution of joint linkages throughout the genome, we first divided the genome into 550-kb bins and counted the number of jointly significant traits at a FDR **≤** 10% in each pair-wise bin (Figure 3A), where simulations demonstrate that the number of bins expected to have three or more two-locus linkages by chance is less than one. Figure 3A indicates that the genomic distribution of joint linkages does not solely follow simple patterns, which is evidence that the “joint” linkage here is meaningful. To test whether similar observations would extend to pairs of linkages on a finer scale, we further divided the genome into 50-kb bins and counted the number of significant joint linkages occurring in each bin. We observed 10 pair-wise bins with three or more traits (Figure 3B), where the number expected by chance is much less than one. This suggests that the same pair of QTL or closely linked QTL contribute to variation in the gene expression levels among all traits falling into any given 50-kb pair-wise bin. Not surprisingly, groups of traits defined by linkage bins possess similar biological functions (Table S2). For example, the 12 traits that jointly link to nearly identical positions on Chromosomes 3 and 8 are predominantly involved in the mating response. The linkage peak on Chromosome 3 maps to the precise location of the yeast mating type locus *MAT*. The parental strains are of opposite mating type, and mating type segregates in the cross. We show elsewhere that variation in the expression of genes in this group is indeed explained by inheritance at *MAT* on Chromosome 3 and at the pheromone response gene *GPA1* on Chromosome 8 [32]. Common biological themes can be assigned to the majority of the remaining clusters including amino acid and mitochondria metabolism (13 traits defined by linkage to regions on Chromosomes 3 and 13), mitochondrial tricarboxylic acid cycle (ten traits defined by linkage to regions on opposite ends of Chromosome 15), and response to stress (five traits defined by linkage to Chromosomes 6 and 10). Table S2 provides a complete list of genes and putative biological functions for the ten pair-wise bins with three or more traits.

Another interesting observation that emerges from the spatial distribution of joint linkages is that distinct groups are connected by a common linkage peak. For example, of the 10 pair-wise bins with three or more linked traits, there are three that share a common linkage to the exact same position on Chromosome 15 (Table S2). Many of the genes in these three groups are localized to the mitochondria, suggesting an important QTL on Chromosome 15 that mediates expression levels for numerous mitochondria related genes. An attractive candidate QTL for this region is *IRA2*, which is a regulator of the RAS-cAMP pathway [33] that is located in both the cytoplasm and mitochondria. More generally, these results intimate that multiple locus mapping of gene expression levels may be useful in reconstructing regulatory networks by identifying shared linkages across traits.

## Discussion

We developed a new, computationally efficient statistical method for simultaneously mapping multiple QTL. Whereas conventional linkage analysis has been widely and successfully applied to study one or very few traits at a time, our method is appropriate for analyzing thousands of phenotypes. Pairs of loci were identified sequentially rather than considering all possible combinations, which was shown to be empirically more powerful. The model used to select pairs of loci included an interaction term allowing for possible epistasis. This sequential approach will of course miss locus pairs with primarily epistatic effects (i.e., little or no main effect for either locus), and these may be biologically interesting or important. Also, we have not included any special modifications to handle the case where two QTL are closely linked, although such modifications are likely possible. Even though it is not likely that two locus models give a complete picture of gene regulation [14], such analyses may still provide valuable information as we have shown here and elsewhere [32]. Since including only two loci may have an adverse effect on power when many QTL affect a trait, it may be helpful to adapt composite interval mapping methods to our approach. However, we were able to observe a number of significant linkages using two QTL models.

A major challenge that our method overcomes is to assign joint significance to the pairs of loci. When identifying linked loci in a sequential manner, it is tempting to apply a readily available significance threshold at each stage. For example, existing *p-*value based FDR methods could have been applied at the first stage to identify a set of significant primary loci. The procedure could then have been repeated on this significant subset to obtain a set of significant secondary loci. Although this may initially appear to be valid, biases are incurred because of the high-dimensional nature of the problem. Specifically, the set of primary loci called significant at the first stage explain a large proportion of variance of their corresponding expression traits, even if the primary loci are false positives. This must be taken into consideration when assessing the significance of secondary loci, which is not the case in simplistic sequential applications of existing *p-*value based methodology. In our approach, we explicitly took into account the sequential selection procedure in order to obtain an overall significance measure of joint linkage.

As technological advances in gene, protein, and metabolite profiling continue to be made, we anticipate that statistical methods such as the one proposed here will provide important insights into the genetic architecture of complex and quantitative traits.

## Materials and Methods

### 

#### Expression measurements

These expression data have recently been reported elsewhere [14]. Briefly, 112 F1 segregants (one from each tetrad) were grown from a cross involving parental strains BY4716, isogenic to the lab strain S288C, and the wild isolate RM11-1a [5,7,14]. RNA was isolated and cDNA was hybridized to microarrays [5,7,14]. Each hybridization was done in the presence of the same BY reference material, and all reported expression values are log_2_(sample/BY reference), averaged over two dye-swapped arrays. Each array [34] assayed 6,216 yeast ORFs, 13 of which were spotted twice, and we did not incorporate special corrections for potential cross-hybridization [35].

#### Genotyping procedure

As previously reported [14], GeneChip Yeast Genome S98 microarrays were purchased from Affymetrix (Santa Clara, California, United States). Genomic DNA was isolated and genotype-calling algorithms were performed as before on all 112 F1 segregants [5]. The resulting genetic map of 3,312 markers covered more than 99% of the genome [5]. When genetic markers are sparse, interval mapping methods [36] can be used to impute mixtures of pseudo-markers in between known observed markers in an attempt to increase power to detect linkage. However, more than 90% of adjacent markers have five or less differences among the 112 progeny, and only 1,226 unique sets of alleles exist among the 3,312 typed. In this case, it sufficed to test for linkage only at these unique sets of alleles. The method proposed here is easily extended to the interval mapping paradigm.

#### Exhaustive 2D test for linkage and epistasis

All pairs of loci were tested for linkage to a given trait based on an F-statistic comparing the least fitted two-locus full model to the null model of no linkage. In order to ease the computational burden, we considered only 613 equally spaced loci and we did not consider any pairs of loci located on the same chromosome. For each trait, the pair of loci with the largest F-statistic was selected. A *p-*value was calculated for each selected pair by using a standard permutation technique [25]: the ordering of the arrays was randomly permuted, and a new maximal F-statistic was recorded for each trait. Five permutations were carried out, and the *p-*value was calculated as the frequency of simulated null F-statistics that exceed the observed statistic. Note that in doing this, the null F-statistics were pooled across traits (giving 5 **×** 6,216 null statistics). This can be justified by noting that the F-statistic is a pivotal statistic and the number of observations (112) is reasonably large. For the test of epistasis, an F-statistic was formed for each pair of loci that compared the full model fit to a purely additive model fit, which directly tests for an interaction between the two loci. For each trait, the pair of loci with the largest F-statistic was selected. In calculating *p-*values, a similar permutation technique was performed that also takes into account the fact that the null model is the additive model [26]. The *p-*values were corrected for multiple testing by employing the FDR [27,28].

#### Comparison between 2D and sequential selection procedures

In order to compare the power of the 2D and sequential selection procedures, the sequential locus selection procedure (described below) was also performed exactly as above, on the same loci, the same null permutations, etc. Therefore, the only aspect compared is the exact procedure used to choose a pair of loci. The *p*-values were obtained from a test against the null hypothesis of no linkage and a test against the null hypothesis of no epistasis. These were compared between the two procedures, and the sequential procedure showed more power in both scenarios (see Results).

#### Sequential selection of locus pairs

For each fixed trait *i,* the primary locus was chosen as the one showing the most single linkage to the trait. Specifically, an F-statistic was calculated for each locus that compares the goodness of fit of the least squares model under the case of no linkage to the least squares model under the case that a single locus is linked. The secondary locus is similarly chosen by fitting a least squares model of trait *i* on its primary locus and each additional locus under the full two-locus model (which includes their additive terms and an interaction term). The locus showing the best improvement in fit, again quantified by a standard F-statistic, is chosen as the secondary locus. Although pairs of loci residing on the same chromosome were not considered in the comparison to the exhaustive 2D search approach, we place no restriction on loci in the main proposed method, i.e., all available loci are considered at each stage of the sequential selection.

#### Calculation of observed and null statistics used to estimate locus-specific linkage probabilities

Let *F_i_*_1_ be the maximal F-statistic corresponding to the primary locus for each trait *i* = 1, … , 6,216. Let *F_i_*_2_ be the maximal F-statistic corresponding to the secondary locus for each trait *i* = 1, … , 6,216. In general, *L* loci may be sequentially selected and F*_ij_* analogously calculated, *j* = 1, … , L.

Statistics from the null distributions were simulated by randomly permuting the ordering of the arrays and calculating a new maximal F-statistic for each trait [25]. For the secondary locus null distribution, the permutations take place within each segregant group corresponding to the primary locus, which takes into account the fact that the null distribution on the secondary locus statistics is calculated under the assumption that the primary locus is truly linked [26]. Five permutations were carried out for each selection stage to yield sets of null statistics *F_i_*_1_^0*b*^ and *F_i_*_2_^0*b*^ for *i* = 1, … , 6,216 and *b* = 1, … , 5. The null F-statistics corresponding to a given locus selection stage were pooled across traits, yielding 5 × 6,216 null statistics. This can be justified again by noting that the F-statistic is an asymptotically pivotal statistic and the number of observations (112) is reasonably large.

#### Nonparametric estimation of locus-specific and joint-linkage probabilities

The observed *F_ij_* and null *F*_*i*1_^0*b*^ are directly used to estimate the locus-specific linkage probabilities. Define ℓ*_ij_* = 1 if the *j*th marker chosen for trait *i* is linked, and ℓ*_ij_* = 0 if no linkage exists; *i* = 1, … , 6,216 and *j* = 1, … , *L*. A standard Bayesian analysis would parameterize a model for the data and also assign prior probabilities to the ℓ*_i_*_1_, ℓ*_i_*_2_, … , ℓ*_i_*_L_. Since there is an enormous amount of data available, we can avoid making some of these assumptions. Let *F_ij_* be the statistic corresponding to the *j*th locus chosen for trait *i*. First, we replace **Pr**(ℓ*_ij_* = 1|ℓ*_i_*_1_ = 1, … , ℓ*_i,j_*_−1_ = 1, Data) with **Pr**(ℓ*_ij_* = 1|ℓ*_i_*_1_ = 1, … , ℓ*_i,j_*_−1_ = 1, *F_ij_*) which may lead to a loss of information at the cost of making less assumptions. The *F_ij_* and F*_ij_* ^0*b*^ are calculated under the assumption that ℓ*_i_*_1_ = 1, … , ℓ*_i,j_*_−1_ = 1 so this is a coherent formulation.

All of the information shown in Figure 2 is not needed in order to simply estimate the locus-specific linkage probabilities. There are essentially only two components that need to be estimated. Take, for example, the calculation of the primary locus probability of linkage, and suppose that the null and alternative distributions of *F_i_*_1_ have probability density functions *g*_0_ and *g*_1_, respectively. Then if π_0_ of the primary loci are not linked and π_1_ are linked, a randomly selected *F_i_*_1_ follows the mixture density g = π_0_ *g*_0_ + π_1_ *g*_1_. (The density functions *g*_0_ and *g*_1_and prior probabilities π_0_ and π_1_ are not assumed to be the same at each locus selection stage. Also, if 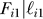 differ between the traits, one can view *g*_0_ and *g*_1_ as the average of these.) According to Bayes theorem, the posterior probability of linkage for the primary locus is



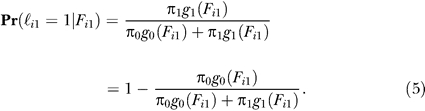



Since *F_i_*_1_ are observations from *g* = π_0_ *g*_0_ + π_1_ *g*_1_ function, and the simulated null 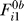 are observations from *g*_0_, these two sets of statistics can be used to estimate the likelihood ratio *g*_0_/*g*, where we define *R*(*F*) = *g*_0_(*F*)/*g*(*F*). Anderson and Blair [37] have shown that with a fixed number of observations from two probability densities, it is valid to estimate their likelihood ratio by performing a logistic regression where, say, the *F_i_*_1_ are called “successes” and the 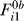 are called “failures.” Methods to perform this logistic regression with a nonparametric link function have been previously developed [37,38]. Using this technique, we form an estimate of the likelihood ratio function denoted by 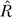. Specifically, the link function is parameterized by a natural cubic spline as previously described [38], with 6,216 knots evenly distributed among all observed and null statistics. A similar procedure has been applied in several applications, for example, in identifying differentially expressed genes [39].

The quantity π_0_ is estimated by



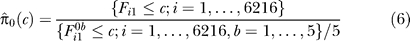



This estimate was originally formulated for use in estimating *p-*value based FDRs [27,28]. It is straightforward to show under our assumptions that the expected value of 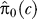 is greater than or equal to π_0_, thus providing a conservative estimate. Adjusting the tuning parameter *c* allows one to balance bias and variance in the estimate. In order to automatically deal with the choice of *c*, we smoothed over a range of *c* using a technique previously described [28]. In this context, another estimate of π_0_ has been suggested as 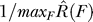 [39]; however, we found this to be much too unstable. For the primary loci we estimate 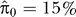, and for the secondary loci 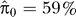. The overall primary locus linkage probability estimate is then



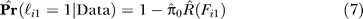



for *i* = 1, . . . , 6,216. The secondary locus linkage probability estimate (and any subsequently selected locus) is formed analogously based on its observed statistics *F_i_*_2_ and simulated null statistics 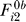. Finally, the two-locus joint-linkage probability is estimated by



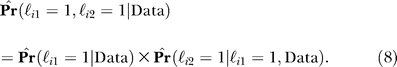



#### FDR estimation

We ranked the traits for significance by the magnitude of the 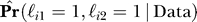 and chose significance cut-offs by calling all trait-locus pair combinations significant that have 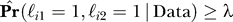 for some λ. Let *S*_λ_ be the set of traits called significant with this threshold and 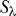 be the number of traits called significant. Defining a trait to be a false discovery if either locus is a false positive, we estimated the FDR by



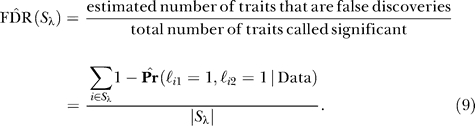



Setting λ = 0.84, we estimate the FDR to be 10%. The estimate can be generalized to *L* loci by simply replacing 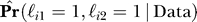 with 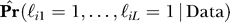. This procedure can also be made more general by noting that any thresholding rule may be used. For example, if one wants to consider traits where both locus-specific linkage probabilities reach a certain level, one may define *S*_λ_ by those traits where 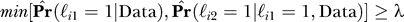. Suppose that one wants to guarantee that 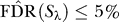, but it is unknown how many loci *L* to choose for each trait. Define 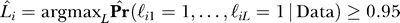; if this is not true for any value set 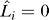. Calling all traits *i* and top 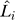 loci jointly linked (among traits with 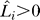), then it follows that 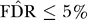 over this set.

Rather than motivating this estimate of the FDR from a model-based Bayesian framework [40,41], we can justify it from the more common frequentist viewpoint. The FDR is usually estimated among multiple hypothesis tests where the null hypothesis is simple (i.e., contains only one parameter value). However, here the null hypothesis consists of the three scenarios where 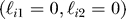, 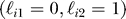, and 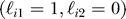. Using previously developed theory [30], it follows that



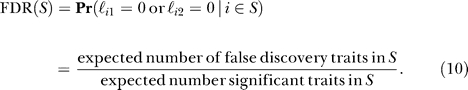



In situations where the FDR can be written in this way, it has been shown in a variety of scenarios that estimates of the form



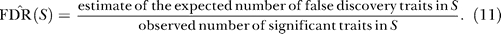



control the FDR as long as the estimated number of expected false discoveries is conservative as the number of traits gets large [42]. Specifically, we can consider the 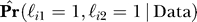 to be random variables and view the numerator and denominator of the estimate to be based on the empirical distribution function of these random variables. It has been shown that as long as the empirical distribution function converges properly, then the above FDR estimate is conservative not only at a fixed threshold, but also at all adaptively chosen thresholds [42]. The main hurdle to overcome beyond this existing theory [42] is to show that 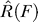 is a consistent estimate of *g*_0_(*F*)/*g*(*F*) in such a way that the convergence of the empirical distribution function is not adversely affected. Nonparametric logistic regression is quite flexible and provides consistent estimates in a fairly general sense as long as the smoothness of the link function decreases at a proper rate [38]. Thus, a reasonably general frequentist justification of our approach based on existing theory appears to be within reach. A battery of simulations (data not shown) indicates that our proposed approach provides reliable significance estimates in a variety of scenarios.

#### Comparison between 1D linkage scan and proposed method

In a traditional 1D linkage scan, a statistic is calculated at each marker and a significance threshold is applied to these in order to find markers showing significant linkage. It is possible that more than one linkage statistic will exceed the threshold. Therefore, one could view this procedure as a multiple locus linkage analysis. We compared this approach to our proposed method by thresholding the two top linkage peaks for each trait. (Peaks were considered to be distinct if they lay on different chromosomes.) A *p-*value was calculated for each trait under the alternative hypothesis that both peaks are true linkages, making the hypothesis test equivalent to ours. Specifically, the *p-*value was defined to be the probability that a statistic exceeded the minimum of the two peaks under the case of no linkage. The *p-*values were then used to estimate the FDR at various significance cut-offs [28]. Since this 1D approach does not take into account any interaction between the two loci, we compared it to our proposed method when using a purely additive model in order to select loci. We found that our method yields 3.3 to 8.7 times more linkages at FDR cut-offs ranging from 1% to 10%.

## Supporting Information

Table S1 Information about the 170 Traits That Possess Joint Linkage at FDR Less Than or Equal to 10% The “*q*-value-Joint” column gives the *q*-value for joint linkage, where a *q*-value is the FDR analog of the *p-*value. In the “Cis” column, zero denotes no *cis* linkage, one denotes *cis* linkage to the primary locus (locus 1), and two denotes *cis* linkage to the secondary locus (locus 2).
(44 KB PDF).

Table S2 Linkages That Cluster according to the Pair-Wise Position of the Two Loci The genome was split into 50-kb pair-wise bins, and the number of significant linkages at FDR less than or equal to 10% falling into each bin was recorded. For any bin containing more than three linkages, the exact marker positions and expression traits are listed below.
(49 KB PDF).

### Accession Numbers

The expression data reported in this paper have been deposited in the Gene Expression Omnibus (GEO) (http://www.ncbi.nlm.nih.gov/geo) database (GSE1990).

## Abbreviations

1D: one-dimensional
2D: two-dimensional
FDR: false discovery rate
kb: kilobase
QTL: quantitative trait loci
