# Supplementary material for: Multiple Locus Linkage Analysis of Genomewide Expression in Yeast
Source: PLoS Biol. 2005 Jul 26;3(8):e267. doi: 10.1371/journal.pbio.0030267 (PMC1180512; doi:10.1371/journal.pbio.0030267)
Supplement: Supplementary file 1 [file pbio.0030267.st001.pdf]

**Supplementary Table 1**. Information on the 170 traits that possess joint linkage at FDR = 10%. The "q-value-Joint" column gives the q-vlaue for joint linkage, where a q-value is the FDR analogue of the p-value. In the "Cis" column, 0 denotes no cis linkage, 1 denotes cis linkage to the primary locus (locus 1), and 2 denotes cis linkage to the secondary locus (locus 2).

| Trait | Chr | Start | Stop | Locus 1 Chr | Locus 1 Pos | Locus 2 Chr | Locus 2 Pos | q-value Joint | Cis |
|---|---|---|---|---|---|---|---|---|---|
| YAL008W | 1 | 136916 | 137512 | 15 | 172654 | 14 | 486861 | 0.085 | 0 |
| YAR020C | 1 | 177021 | 176854 | 4 | 722072 | 15 | 136326 | 0.0699 | 0 |
| YAR033W | 1 | 188101 | 188805 | 7 | 404437 | 1 | 185880 | 0.0282 | 2 |
| YAR047C | 1 | 201780 | 201460 | 1 | 199762 | 5 | 395442 | 0.0734 | 1 |
| YBR068C | 2 | 375687 | 373858 | 3 | 90986 | 13 | 67735 | 0.0351 | 0 |
| YBR069C | 2 | 378430 | 376571 | 2 | 376562 | 5 | 350744 | 0.0564 | 1 |
| YBR083W | 2 | 409163 | 410623 | 8 | 111686 | 2 | 405289 | 0.0226 | 2 |
| YBR166C | 2 | 571195 | 569837 | 2 | 565216 | 3 | 105042 | 0.0883 | 1 |
| YBR281C | 2 | 767602 | 764966 | 2 | 771132 | 15 | 179409 | 0.0977 | 1 |
| YCL073C | 3 | 8326 | 6479 | 12 | 501030 | 11 | 651631 | 0.0559 | 0 |
| YCL066W | 3 | 13282 | 13809 | 3 | 201166 | 12 | 423789 | 0.1008 | 0 |
| YCL065W | 3 | 13751 | 14119 | 3 | 201166 | 8 | 111679 | 0.0726 | 0 |
| YCL064C | 3 | 16880 | 15798 | 13 | 49969 | 10 | 69578 | 0.0998 | 0 |
| YCR039C | 3 | 200173 | 199541 | 3 | 201166 | 8 | 80036 | 0.0708 | 1 |
| YCR075C | 3 | 248811 | 248029 | 14 | 449639 | 15 | 172654 | 0.0928 | 0 |
| YCR096C | 3 | 293534 | 293175 | 3 | 201166 | 8 | 80036 | 0.0963 | 0 |
| YCR097W | 3 | 294236 | 294287 | 3 | 201166 | 7 | 557233 | 0.0854 | 0 |
| YCR106W | 3 | 310954 | 313452 | 12 | 1056103 | 11 | 590 | 0.0585 | 0 |
| YDL217C | 4 | 68607 | 67984 | 4 | 74576 | 14 | 486861 | 0.0663 | 1 |
| YDL181W | 4 | 135180 | 135437 | 15 | 172654 | 15 | 563943 | 0.0595 | 0 |
| YDL174C | 4 | 147590 | 145827 | 12 | 671271 | 4 | 143910 | 0.0917 | 2 |
| YDL127W | 4 | 234927 | 235853 | 8 | 111686 | 4 | 192248 | 0.0686 | 0 |
| YDL039C | 4 | 382330 | 381983 | 4 | 369366 | 5 | 350744 | 0.0413 | 0 |
| YDL038C | 4 | 384078 | 382327 | 4 | 369366 | 5 | 350744 | 0.0484 | 0 |
| YDL037C | 4 | 385584 | 384598 | 4 | 369366 | 5 | 350744 | 0.062 | 0 |
| YDR043C | 4 | 543366 | 542671 | 2 | 548401 | 5 | 395442 | 0.1015 | 0 |
| YDR044W | 4 | 546639 | 547625 | 12 | 660992 | 5 | 350744 | 0.0405 | 0 |
| YDR112W | 4 | 679539 | 679847 | 3 | 76127 | 13 | 49969 | 0.1001 | 0 |
| YDR111C | 4 | 679759 | 678236 | 3 | 76127 | 13 | 49969 | 0.064 | 0 |
| YDR133C | 4 | 721292 | 720957 | 4 | 722072 | 15 | 172654 | 0.0296 | 1 |
| YDR134C | 4 | 721476 | 721066 | 4 | 722072 | 15 | 172654 | 0.0397 | 1 |
| YDR148C | 4 | 755061 | 753670 | 15 | 172654 | 15 | 563943 | 0.0739 | 0 |
| YDR178W | 4 | 817945 | 818490 | 15 | 563943 | 15 | 172654 | 0.1026 | 0 |
| YDR262W | 4 | 993128 | 993946 | 4 | 975086 | 2 | 548401 | 0.0446 | 0 |
| YDR297W | 4 | 1056545 | 1057594 | 4 | 1041331 | 12 | 660992 | 0.0548 | 0 |
| YDR320C | 4 | 1108096 | 1106090 | 4 | 1109144 | 15 | 141627 | 0.076 | 1 |
| YEL038W | 5 | 80420 | 81145 | 5 | 79650 | 15 | 154243 | 0.0949 | 1 |
| YER001W | 5 | 153519 | 155807 | 15 | 141627 | 2 | 562412 | 0.096 | 0 |
| YER044C | 5 | 238015 | 237569 | 12 | 660992 | 5 | 272256 | 0.0529 | 0 |
| YER073W | 5 | 304027 | 305589 | 3 | 90986 | 13 | 67735 | 0.0542 | 0 |
| YER082C | 5 | 325932 | 324268 | 2 | 548401 | 4 | 192248 | 0.0974 | 0 |
| YER139C | 5 | 451238 | 450558 | 5 | 442538 | 15 | 179409 | 0.0823 | 1 |
| YFL061W | 6 | 9545 | 10222 | 10 | 698149 | 6 | 17387 | 0.043 | 2 |
| YFL060C | 6 | 10969 | 10301 | 10 | 698149 | 6 | 17387 | 0.0369 | 2 |
| YFL059W | 6 | 11363 | 12259 | 6 | 5852 | 10 | 698149 | 0.0743 | 1 |
| YFL056C | 6 | 15431 | 14793 | 6 | 17387 | 10 | 23514 | 0.0331 | 1 |
| YFL055W | 6 | 17004 | 18680 | 10 | 23514 | 6 | 17387 | 0.0092 | 2 |
| YFL054C | 6 | 22787 | 20847 | 6 | 17387 | 10 | 23514 | 0.0178 | 1 |
| YFL053W | 6 | 23423 | 25198 | 10 | 34092 | 6 | 17387 | 0.0156 | 2 |
| YFL052W | 6 | 28232 | 29629 | 10 | 23514 | 6 | 17387 | 0.0777 | 0 |
| YFL027C | 6 | 81910 | 80417 | 3 | 201166 | 8 | 71988 | 0.1036 | 0 |
| YFL026W | 6 | 82578 | 83873 | 3 | 201166 | 8 | 80036 | 0.0773 | 0 |
| YFR026C | 6 | 206245 | 205736 | 8 | 111686 | 6 | 208330 | 0.1049 | 2 |
| YGL258W | 7 | 11110 | 11730 | 10 | 329085 | 12 | 35970 | 0.0891 | 0 |
| YGL256W | 7 | 14910 | 16307 | 10 | 325500 | 12 | 35970 | 0.0865 | 0 |
| YGL208W | 7 | 97342 | 98589 | 7 | 106500 | 15 | 172654 | 0.061 | 1 |
| YGL204C | 7 | 112009 | 111704 | 7 | 106500 | 4 | 573400 | 0.0765 | 1 |
| YGL191W | 7 | 144814 | 145203 | 12 | 671271 | 15 | 563943 | 0.0834 | 0 |
| YGL174W | 7 | 174551 | 175351 | 7 | 167587 | 14 | 449639 | 0.0615 | 1 |
| YGL138C | 7 | 249536 | 248499 | 3 | 90986 | 9 | 258738 | 0.047 | 0 |
| YGL090W | 7 | 343322 | 344587 | 3 | 201166 | 8 | 111679 | 0.0233 | 0 |
| YGL089C | 7 | 345156 | 344794 | 3 | 201166 | 8 | 111686 | 0.0876 | 0 |
| YGL032C | 7 | 436838 | 436575 | 3 | 201166 | 8 | 111686 | 0.0274 | 0 |
| YGL009C | 7 | 478657 | 476318 | 3 | 90986 | 13 | 67735 | 0.059 | 0 |
| YGL001C | 7 | 496506 | 495457 | 12 | 660992 | 7 | 488370 | 0.0769 | 2 |
| YGR007W | 7 | 506973 | 507944 | 12 | 660992 | 2 | 548401 | 0.0981 | 0 |
| YGR040W | 7 | 575402 | 576508 | 7 | 557233 | 2 | 562412 | 0.0497 | 0 |
| YGR049W | 7 | 591318 | 591881 | 12 | 660992 | 7 | 592983 | 0.012 | 2 |
| YGR050C | 7 | 592446 | 592090 | 7 | 592983 | 12 | 674651 | 0.0462 | 1 |
| YGR079W | 7 | 640724 | 641836 | 2 | 537314 | 15 | 158978 | 0.088 | 0 |
| YGR108W | 7 | 703640 | 705055 | 9 | 141014 | 13 | 328865 | 0.079 | 0 |
| YGR126W | 7 | 745840 | 746532 | 7 | 795526 | 10 | 67350 | 0.1032 | 0 |
| YGR146C | 7 | 784863 | 784228 | 3 | 79091 | 8 | 111686 | 0.0672 | 0 |
| YGR163W | 7 | 827557 | 828582 | 2 | 565216 | 3 | 90986 | 0.0605 | 0 |
| YGR235C | 7 | 962064 | 961363 | 12 | 660992 | 9 | 296453 | 0.1029 | 0 |
| YHR007C | 8 | 121677 | 120085 | 8 | 98516 | 12 | 660992 | 0.032 | 0 |
| YHR016C | 8 | 138400 | 138233 | 15 | 172654 | 14 | 486861 | 0.0984 | 0 |
| YHR072W | 8 | 239099 | 241294 | 12 | 660992 | 8 | 248252 | 0.0872 | 2 |
| YHR080C | 8 | 266839 | 262802 | 15 | 172654 | 2 | 372476 | 0.0421 | 0 |
| YHR115C | 8 | 341361 | 340111 | 8 | 316306 | 2 | 537314 | 0.1039 | 0 |
| YHR162W | 8 | 423074 | 423463 | 13 | 42129 | 3 | 79091 | 0.0846 | 0 |
| YHR178W | 8 | 459298 | 461529 | 3 | 201166 | 16 | 535078 | 0.0752 | 0 |
| YHR198C | 8 | 497279 | 496314 | 15 | 172654 | 14 | 449639 | 0.0717 | 0 |
| YHR218W | 8 | 558615 | 558713 | 12 | 1056097 | 4 | 1501558 | 0.0887 | 0 |

| | | | | | | | | | |
|---|---|---|---|---|---|---|---|---|---|
| YIL169C | 9 | 26106 | 23119 | 15 | 8341 | 2 | 554641 | 0.1005 | 0 |
| YIL121W | 9 | 132241 | 133869 | 12 | 677962 | 3 | 90986 | 0.0218 | 0 |
| YIL119C | 9 | 137874 | 136651 | 13 | 328865 | 9 | 139479 | 0.0454 | 2 |
| YIL087C | 9 | 200116 | 199643 | 9 | 199116 | 15 | 172654 | 0.0341 | 1 |
| YIL082W | 9 | 205632 | 206504 | 7 | 708912 | 9 | 258738 | 0.0894 | 0 |
| YIL080W | 9 | 206482 | 206482 | 8 | 111686 | 9 | 190988 | 0.0935 | 0 |
| YIL082W-A | 9 | 206483 | 206483 | 7 | 708912 | 8 | 111686 | 0.051 | 0 |
| YIL015W | 9 | 322340 | 324103 | 3 | 201166 | 8 | 80036 | 0.0253 | 0 |
| YIR017C | 9 | 384116 | 383553 | 3 | 75021 | 10 | 387893 | 0.1046 | 0 |
| YIR019C | 9 | 393672 | 389569 | 5 | 395442 | 9 | 399057 | 0.0649 | 2 |
| YJL171C | 10 | 100888 | 99698 | 10 | 87113 | 3 | 201166 | 0.058 | 0 |
| YJL170C | 10 | 101695 | 101144 | 3 | 201166 | 8 | 111686 | 0.0309 | 0 |
| YJL150W | 10 | 137041 | 137343 | 3 | 201166 | 2 | 256896 | 0.0438 | 0 |
| YJL108C | 10 | 218773 | 217622 | 10 | 216786 | 12 | 660992 | 0.0635 | 1 |
| YJR004C | 10 | 444775 | 442823 | 3 | 201166 | 8 | 111686 | 0.0209 | 0 |
| YJR015W | 10 | 462635 | 464167 | 10 | 461201 | 3 | 105042 | 0.0654 | 1 |
| YJR016C | 10 | 466122 | 464365 | 3 | 90986 | 13 | 67735 | 0.0625 | 0 |
| YJR077C | 10 | 578104 | 577169 | 15 | 563943 | 15 | 172654 | 0.0721 | 0 |
| YJR104C | 10 | 622927 | 622463 | 10 | 628109 | 15 | 179409 | 0.06 | 1 |
| YJR113C | 10 | 638890 | 638147 | 2 | 517365 | 14 | 449639 | 0.097 | 0 |
| YJR120W | 10 | 647041 | 647391 | 15 | 563943 | 15 | 172654 | 0.0806 | 0 |
| YJR127C | 10 | 662974 | 658832 | 10 | 646911 | 15 | 172654 | 0.0827 | 0 |
| YJR148W | 10 | 705659 | 706789 | 13 | 49969 | 3 | 105042 | 0.0536 | 0 |
| YKL217W | 11 | 22234 | 24084 | 11 | 45334 | 2 | 513722 | 0.092 | 0 |
| YKL140W | 11 | 180784 | 182430 | 11 | 180221 | 12 | 681956 | 0.036 | 1 |
| YKL135C | 11 | 188647 | 186467 | 11 | 194611 | 8 | 80036 | 0.0819 | 1 |
| YKL120W | 11 | 216990 | 217964 | 3 | 90986 | 13 | 46077 | 0.0504 | 0 |
| YKL096W | 11 | 260777 | 261496 | 11 | 261779 | 15 | 172654 | 0.0668 | 1 |
| YKL085W | 11 | 278768 | 279772 | 15 | 172654 | 15 | 563943 | 0.0991 | 0 |
| YKL043W | 11 | 356393 | 357493 | 11 | 356622 | 5 | 350744 | 0.0902 | 1 |
| YKL040C | 11 | 361472 | 360702 | 5 | 395442 | 15 | 172654 | 0.0523 | 0 |
| YKL030W | 11 | 382141 | 382746 | 3 | 76127 | 9 | 243418 | 0.0785 | 0 |
| YKL008C | 11 | 428194 | 426938 | 12 | 660992 | 2 | 554641 | 0.0264 | 0 |
| YKR051W | 11 | 530588 | 531844 | 11 | 527135 | 12 | 660992 | 0.0869 | 1 |
| YKR080W | 11 | 590037 | 590999 | 11 | 590637 | 13 | 49969 | 0.0477 | 1 |
| YKR085C | 11 | 599323 | 598736 | 11 | 599170 | 14 | 486861 | 0.0802 | 1 |
| YKR105C | 11 | 660102 | 658354 | 11 | 651631 | 16 | 825431 | 0.1019 | 1 |
| YLL028W | 12 | 84803 | 86563 | 15 | 146302 | 12 | 86369 | 0.0554 | 2 |
| YLL023C | 12 | 98835 | 97996 | 12 | 99261 | 15 | 172654 | 0.0659 | 1 |
| YLR050C | 12 | 246073 | 245588 | 12 | 254693 | 12 | 660992 | 0.0239 | 1 |
| YLR109W | 12 | 368782 | 369312 | 13 | 255525 | 2 | 562412 | 0.081 | 0 |
| YLR118C | 12 | 385409 | 384726 | 14 | 486861 | 12 | 381332 | 0.0956 | 2 |
| YLR179C | 12 | 514715 | 514110 | 12 | 514835 | 12 | 748651 | 0.0938 | 1 |
| YLR355C | 12 | 839252 | 838065 | 13 | 46077 | 3 | 100213 | 0.0794 | 0 |
| YLR382C | 12 | 884751 | 882067 | 14 | 449639 | 15 | 154243 | 0.0713 | 0 |
| YML123C | 13 | 25801 | 24038 | 13 | 40477 | 10 | 36810 | 0.057 | 0 |
| YML002W | 13 | 264541 | 266754 | 15 | 563943 | 15 | 172654 | 0.1022 | 0 |
| YMR008C | 13 | 282584 | 280590 | 13 | 275164 | 15 | 179409 | 0.0942 | 1 |
| YMR022W | 13 | 318679 | 319176 | 13 | 328865 | 2 | 681442 | 0.0379 | 1 |
| YMR081C | 13 | 431094 | 430078 | 2 | 507554 | 4 | 192248 | 0.0953 | 0 |
| YMR096W | 13 | 458407 | 459300 | 3 | 90986 | 13 | 67735 | 0.0967 | 0 |
| YMR108W | 13 | 484083 | 486146 | 3 | 90986 | 13 | 42129 | 0.0677 | 0 |
| YMR145C | 13 | 556474 | 554792 | 12 | 660992 | 15 | 563943 | 0.0747 | 0 |
| YMR306C-A | 13 | 886571 | 886182 | 13 | 885665 | 10 | 731761 | 0.0994 | 1 |
| YMR321C | 13 | 917894 | 917577 | 13 | 922258 | 3 | 75021 | 0.0898 | 1 |
| YNL194C | 14 | 273614 | 272709 | 15 | 172654 | 2 | 376562 | 0.0946 | 0 |
| YNL169C | 14 | 317671 | 316169 | 2 | 537314 | 3 | 100213 | 0.0861 | 0 |
| YNL078W | 14 | 479767 | 480990 | 2 | 565216 | 8 | 111686 | 0.049 | 0 |
| YNL065W | 14 | 503723 | 505483 | 5 | 116389 | 14 | 502406 | 0.0695 | 2 |
| YNL034W | 14 | 570476 | 572314 | 14 | 616445 | 5 | 116389 | 0.0681 | 0 |
| YNL018C | 14 | 601773 | 599935 | 14 | 578484 | 5 | 116389 | 0.0196 | 0 |
| YNR019W | 14 | 665339 | 667267 | 12 | 660992 | 1 | 42348 | 0.0931 | 0 |
| YNR052C | 14 | 720647 | 719346 | 14 | 502406 | 12 | 611832 | 0.069 | 0 |
| YOL104C | 15 | 117453 | 116395 | 15 | 132423 | 2 | 636645 | 0.0815 | 0 |
| YOL101C | 15 | 127919 | 126981 | 1 | 42348 | 7 | 405999 | 0.0243 | 0 |
| YOR051C | 15 | 426085 | 424847 | 15 | 436372 | 2 | 548401 | 0.0781 | 0 |
| YOR142W | 15 | 593057 | 594046 | 15 | 563943 | 15 | 172654 | 0.0909 | 0 |
| YOR148C | 15 | 609197 | 608640 | 12 | 674651 | 7 | 592983 | 0.0842 | 0 |
| YOR153W | 15 | 619840 | 624375 | 7 | 460150 | 14 | 486861 | 0.0838 | 0 |
| YOR162C | 15 | 641992 | 639560 | 3 | 201166 | 8 | 111679 | 0.0575 | 0 |
| YOR171C | 15 | 653884 | 652010 | 2 | 405289 | 12 | 635380 | 0.0988 | 0 |
| YOR221C | 15 | 757737 | 757537 | 3 | 76127 | 13 | 28610 | 0.0704 | 0 |
| YOR263C | 15 | 819161 | 818754 | 2 | 548401 | 5 | 395442 | 0.1012 | 0 |
| YOR317W | 15 | 909338 | 911440 | 15 | 172654 | 12 | 671271 | 0.0913 | 0 |
| YOR388C | 15 | 1072919 | 1071789 | 10 | 43261 | 15 | 1069040 | 0.083 | 2 |
| YPL276W | 16 | 17948 | 18385 | 10 | 43261 | 15 | 1069040 | 0.0388 | 0 |
| YPL275W | 16 | 18369 | 19079 | 10 | 61620 | 2 | 581702 | 0.0857 | 0 |
| YPL271W | 16 | 30079 | 30267 | 15 | 563943 | 15 | 172654 | 0.0645 | 0 |
| YPL177C | 16 | 213961 | 213041 | 5 | 395442 | 5 | 116389 | 0.073 | 0 |
| YPL140C | 16 | 289033 | 287513 | 14 | 449639 | 16 | 280629 | 0.0517 | 2 |
| YPL064C | 16 | 429613 | 428708 | 12 | 660992 | 16 | 429185 | 0.0798 | 2 |
| YPL061W | 16 | 432585 | 434087 | 13 | 128290 | 12 | 634227 | 0.0924 | 0 |
| YPL057C | 16 | 453054 | 451906 | 16 | 462646 | 1 | 52357 | 0.0906 | 1 |
| YPL004C | 16 | 551654 | 550629 | 15 | 172654 | 15 | 563943 | 0.1043 | 0 |
| YPR150W | 16 | 830995 | 831516 | 12 | 660992 | 12 | 955920 | 0.0756 | 0 |
| YPR192W | 16 | 921856 | 922773 | 16 | 927503 | 15 | 172654 | 0.063 | 1 |
