# Supplementary material for: Multiple Locus Linkage Analysis of Genomewide Expression in Yeast
Source: PLoS Biol. 2005 Jul 26;3(8):e267. doi: 10.1371/journal.pbio.0030267 (PMC1180512; doi:10.1371/journal.pbio.0030267)
Supplement: Supplementary file 2 [file pbio.0030267.st002.pdf]

Supplementary Table 2. Linkages that cluster according to the pair-wise position of the two loci. The genome was split into 50 kb pair-wise bins and the number of significant linkages at FDR = 10% falling into each bin was recorded. For any bin containing more than three linkages, the exact marker positions and expression traits are listed below.

Group	Trait	Chr	Pos_start	Pos_end	Locus 1 Chr	Locus 1 Pos	Locus 2 Chr	Locus 2 Pos	Joint FDR	Cis	Putative Group Function
1	YFL027C	6	81910	80417	3	201166	8	71988	0.1036	0	Mating
1	YCR039C	3	200173	199541	3	201166	8	80036	0.0708	1	
1	YCR096C	3	293534	293175	3	201166	8	80036	0.0963	0	
1	YFL026W	6	82578	83873	3	201166	8	80036	0.0773	0	
1	YIL015W	9	322340	324103	3	201166	8	80036	0.0253	0	
1	YGL032C	7	436838	436575	3	201166	8	111686	0.0274	0	
1	YGL089C	7	345156	344794	3	201166	8	111686	0.0876	0	
1	YGL090W	7	343322	344587	3	201166	8	111679	0.0233	0	
1	YCL065W	3	13751	14119	3	201166	8	111679	0.0726	0	
1	YJL170C	10	101695	101144	3	201166	8	111686	0.0309	0	
1	YJR004C	10	444775	442823	3	201166	8	111686	0.0209	0	
1	YOR162C	15	641992	639560	3	201166	8	111679	0.0575	0	
2	YOR221C	15	757737	757537	3	76127	13	28610	0.0704	0	Amino Acid and Mitochondria Metabolism
2	YDR111C	4	679759	678236	3	76127	13	49969	0.064	0	
2	YDR112W	4	679539	679847	3	76127	13	49969	0.1001	0	
2	YHR162W	8	423074	423463	3	79091	13	42129	0.0846	0	
2	YMR108W	13	484083	486146	3	90986	13	42129	0.0677	0	
2	YKL120W	11	216990	217964	3	90986	13	46077	0.0504	0	
2	YER073W	5	304027	305589	3	90986	13	67735	0.0542	0	
2	YBR068C	2	375687	373858	3	90986	13	67735	0.0351	0	
2	YMR096W	13	458407	459300	3	90986	13	67735	0.0967	0	
2	YJR016C	10	466122	464365	3	90986	13	67735	0.0625	0	
2	YGL009C	7	478657	476318	3	90986	13	67735	0.059	0	
2	YLR355C	12	839252	838065	3	100213	13	46077	0.0794	0	
2	YJR148W	10	705659	706789	3	105042	13	49969	0.0536	0	
3	YPL004C	16	551654	550629	15	172654	15	563943	0.1043	0	Mitochondrial TCA
3	YKL085W	11	278768	279772	15	172654	15	563943	0.0991	0	
3	YDL181W	4	135180	135437	15	172654	15	563943	0.0595	0	
3	YDR148C	4	755061	753670	15	172654	15	563943	0.0739	0	
3	YDR178W	4	817945	818490	15	172654	15	563943	0.1026	0	
3	YOR142W	15	593057	594046	15	172654	15	563943	0.0909	0	
3	YML002W	13	264541	266754	15	172654	15	563943	0.1022	0	
3	YJR120W	10	647041	647391	15	172654	15	563943	0.0806	0	
3	YJR077C	10	578104	577169	15	172654	15	563943	0.0721	0	
3	YPL271W	16	30079	30267	15	172654	15	563943	0.0645	0	
4	YHR016C	8	138400	138233	15	172654	14	486861	0.0984	0	Mitochondria
4	YAL008W	1	136916	137512	15	172654	14	486861	0.085	0	
4	YHR198C	8	497279	496314	15	172654	14	449639	0.0717	0	
4	YCR075C	3	248811	248029	15	172654	14	449639	0.0928	0	
4	YLR382C	12	884751	882067	15	154243	14	449639	0.0713	0	
5	YFL052W	6	28232	29629	10	23514	6	17387	0.0777	0	Response To Stress
5	YFL055W	6	17004	18680	10	23514	6	17387	0.0092	1	
5	YFL054C	6	22787	20847	10	23514	6	17387	0.0178	1	
5	YFL056C	6	15431	14793	10	23514	6	17387	0.0331	1	
5	YFL053W	6	23423	25198	10	34092	6	17387	0.0156	1	
6	YGR079W	7	640724	641836	15	158978	2	537314	0.088	0	Unknown
6	YHR080C	8	266839	262802	15	172654	2	372476	0.0421	0	
6	YNL194C	14	273614	272709	15	172654	2	376562	0.0946	0	
7	YDR133C	4	721292	720957	4	722072	15	172654	0.0296	1	Heterogeneous
7	YAR020C	1	177021	176854	4	722072	15	136326	0.0699	0	
7	YDR134C	4	721476	721066	4	722072	15	172654	0.0397	1	
7	YDR320C	4	1108096	1106090	4	1109144	15	141627	0.076	1	
8	YDL039C	4	382330	381983	4	369366	5	350744	0.0413	0	Cell Membrane
8	YDL038C	4	384078	382327	4	369366	5	350744	0.0484	0	
8	YDL037C	4	385584	384598	4	369366	5	350744	0.062	0	
9	YFL060C	6	10969	10301	10	698149	6	17387	0.0369	1	Diauxic Shift
9	YFL061W	6	9545	10222	10	698149	6	17387	0.043	1	
9	YFL059W	6	11363	12259	10	698149	6	52	0.0743	0	
10	YOR148C	15	609197	608640	12	674651	7	592983	0.0842	0	Heterogeneous
10	YGR049W	7	591318	591881	12	660992	7	592983	0.012	1	
10	YGR050C	7	592446	592090	12	674651	7	592983	0.0462	1	
